# A universal incision for robot-assisted thoracic surgery

**DOI:** 10.3389/fsurg.2022.965453

**Published:** 2022-08-23

**Authors:** Jia Jiao, Jinbao Guo, Jia Zhao, Xiangnan Li, Ming Du

**Affiliations:** ^1^ Department of Thoracic Surgery and Lung Transplantation, First Affiliated Hospital of Zhengzhou University, Zhengzhou, China; ^2^ Department of Cardiothoracic Surgery, The First Affiliated Hospital of Chongqing Medical University, Chongqing, China

**Keywords:** robot-assisted, minimally invasive thoracic surgery, lobectomy, segmentectomy, esophagectomy, mediastinal mass

## Abstract

**Objective:**

This paper aimed to design and explore the versatility of the incision for the robot-assisted thoracic surgery.

**Methods:**

The concept of universal incision was designed and put forward. The clinical data of 342 cases of robot-assisted thoracic surgery were summarized, including sex, age, clinical diagnosis, operative method, operative time, conversion to thoracotomy, intraoperative blood loss, number of lymph node dissections, postoperative hospital stays, postoperative pathology, and postoperative complications of the patients.

**Results:**

The 342 cases of robot-assisted surgery included 178 pulmonary surgery cases (94 lobectomy cases, 75 segmentectomy cases, 6 wedge resection cases, and 3 sleeve lobectomy cases), 112 esophageal surgery cases (107 McKeown approach cases and 5 esophageal leiomyoma resection cases), and 52 mediastinal tumor cases (42 anterior mediastinum cases and 10 posterior mediastinum cases). Among these, two cases were converted to thoracotomy (both esophageal cases), and the rest were successful with no massive intraoperative bleeding and no perioperative death.

**Conclusion:**

The universal incision of robot-assisted thoracic surgery is safe and feasible and is suitable for most cases of thoracic surgery.

## Introduction

At the end of the last century, the extensive development of thoracoscopic surgery brought thoracic surgery into the era of minimally invasive surgery. In the last 10 years, robot-assisted thoracic surgery (RATS) has developed rapidly. The Da Vinci Surgical System, which specializes in fine operations such as a high-definition, three-dimensional view, and articulating EndoWrist instruments, has made up for the deficiency of thoracoscopic surgery (1, 2). However, the selection of the incisions for RATS is diverse and has not been unified. Since the Da Vinci Si Robot Surgical System was installed in our hospital in 2016, more than 300 robot-assisted thoracic surgeries have been completed, and some preliminary experience has been accumulated. Currently, a retrospective analysis and summary are made on the case data of robot-assisted surgery in the thoracic surgery department of our hospital to explore the universal incision of RATS.

## Research methods

### General clinical data

The clinical data of 342 patients undergoing RATS in the Thoracic Surgery Department of the First Affiliated Hospital of Chongqing Medical University and the First Affiliated Hospital of Zhengzhou University from April 2016 to September 2021 were analyzed, including sex, age, clinical diagnosis, operation method, operation time, transfer to thoracotomy, intraoperative blood loss, number of lymph node dissections, and postoperative complications.

Postoperative complications mainly included active thoracic bleeding, pulmonary infection, atelectasis, chylothorax, thoracic infection, wound healing, esophagogastric anastomotic fistula, diaphragmatic hernia, and recurrent laryngeal nerve palsy.

### Surgical methods

#### Surgical position and anesthesia intubation

The patient was placed in the lateral decubitus position, and single-lung anesthesia was administered *via* double-lumen endotracheal intubation (for pulmonary surgery) or single-lumen endotracheal intubation and artificial pneumothorax with a CO_2_ pressure of 8 mmHg (for esophagus and mediastinal tumor surgery). Abdominal and neck operations for patients with esophageal cancer were performed through the McKeown approach, with the patients’ head tilted to the right side with high shoulder pads.

#### Incision selection

**Figure 1:**
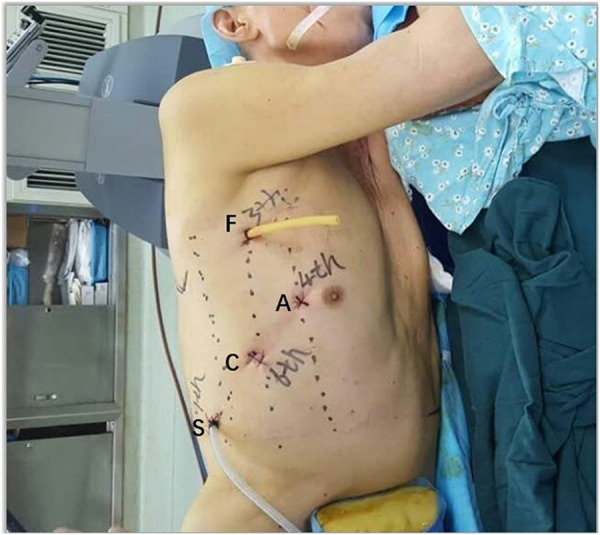
Port placement for the Da Vinci Si System using three robotic arms (thoracic cavity). C, camera port; A, assistant port; F, first robotic arm; S, second robotic arm.

Four-port incisions were made at the positions indicated in Figure 1. A 10 mm port in the sixth intercostal space (ICS) in the midaxillary line was placed as the camera port. The other two incisions were placed at the midaxillary axillary line in the third ICS for the first robotic arm and at the subscapular line in the ninth ICS for the second robotic arm. The assistant port (12 mm trocar for esophagus and mediastinum tumor surgery or extended to a 3-cm incision for pulmonary surgery) was placed at the anterior axillary line in the fourth ICS. These incisions were standard and suitable for all thoracic surgeries, except for the tumor in the anterior mediastinum. When the tumor was located in the anterior mediastinum, the incisions for the second robotic arm were placed at the anterior axillary line in the sixth ICS, and the assistant port was placed at the posterior axillary line in the eighth ICS.

**Figure 2:**
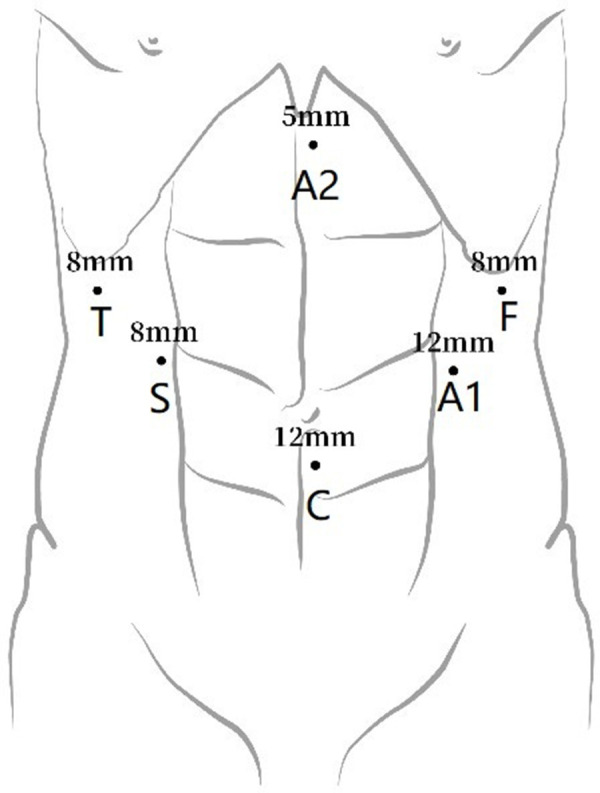
Port placement for abdominal robot-assisted minimally invasive esophagectomy. C, camera port; A1, assistant port 1; A2, assistant port 2; F, first robotic arm; S, second robotic arm; T: third robotic arm.

Abdominal incisions for the patients with esophageal cancer undergoing the McKeown approach: the first, second, and third arms were selected for abdominal operation. The incisions were as follows: the camera port was placed above the level of the umbilicus (12 mm trocar); the incisions for the first/third robotic arm were selected at the left/right middle clavicular line and at the left/right costal margins; and the incisions for the second robotic arm were placed at the right midclavicular line and at the umbilical level. Two other 8-mm assistant incisions were then placed as follows: each at the left middle clavicular line and at the midclavicular umbilical level and another below the xiphoid process, as shown in Figure 2.

#### Device selection

The Da Vinci Si Robot Surgical System (Intuitive Surgical, Sunnyvale, CA, USA) was brought into the field, coming over the patient's head. In the thoracic operation, we used robotic instruments as follows: the first robotic arm was used for the permanent cautery hook and the second robotic arm was used for the fenestrated bipolar forceps. When the left and right recurrent laryngeal nerve chain lymph nodes were dissected in esophageal surgery, the first robotic arm could be temporarily replaced by Maryland bipolar forceps. For the abdominal part of esophageal surgery, we used robotic instruments as follows: the first robotic arm Harmonic ACE was used for the curved shears, the second robotic arm was used for the fenestrated bipolar forceps, and the third robotic arm was used for the Cadiere forceps (mainly used to expose the liver).

#### Surgical methods

Lobectomy and segmentectomy: All patients underwent single-direction thoracoscopic anatomic pulmonary surgery as reported (3, 4). Systemic mediastinal lymph node dissection was performed for patients with invasive lung cancer (Station 5, 6, 7, 8, and 9 lymph nodes for left lung cancer and Station 2, 4, 7, 8, and 9 lymph nodes for right lung cancer).

Esophagectomy: All patients underwent the traditional McKeown approach, which includes thoracic esophageal dissection, abdominal mobilization of the gastric conduit, and cervical anastomosis (5). Lymph nodes of the left and right recurrent laryngeal nerve chains were dissected during the operation.

Mediastinal tumor resection: The tumor was removed completely along its outer membrane.

#### Statistical methods

The SPSS 22.0 statistical software package was used for statistical analysis. Clinical and pathological characteristics were described as the mean ± standard deviation for continuous variables and frequencies (%) for categorical variables.

## Results

### General Information

The 342 robot-assisted surgeries included 178 pulmonary surgery cases (94 lobectomy cases, 75 segmentectomy cases, 6 wedge resection cases, and 3 sleeve lobectomy cases), 112 esophageal surgery cases (107 McKeown approach cases and 5 esophageal leiomyoma resection cases), and 52 mediastinal tumor cases (42 anterior mediastinum cases and 10 posterior mediastinum cases). The general information is detailed in Table 1.

**Table 1:** The general characteristics of 342 patients.

	Pulmonary	Esophagus	Mediastinum
Cases	178	112	52
Gender (male/female)	85/93	72/40	29/23
Age	59	62	49
Lesion location			
Right upper lung	68	Esophageal cancer 107	Anterior mediastinum 42 Posterior mediastinum 10
Right middle lung	16		
Right lower lung	32	Esophageal leiomyoma 5	
Left upper lung	27		
Left lower lung	35		
Surgery types		McKeown approach 107	
Lobectomy	94		
Segmentectomy	75	Leiomyoma resection 5	
Wedge resection	6		
Sleeve lobectomy	3		

### Perioperative data

The average docking time of the 342 patients experiencing robot-assisted surgeries was 7.7 ± 3.3 min, with 2 patients transferred to thoracotomy (both esophageal cases) and the rest successfully completed with no intraoperative massive bleeding. The mean numbers of harvested lymph nodes in the pulmonary group and esophageal group were 15.5 ± 4.9 and 25.3 ± 6.5, respectively. The mean days of postoperative hospital stay in the pulmonary group, esophagus group, and mediastinum group were 6 ± 3, 16 ± 9, and 5 ± 2, respectively. Pneumonia occurred in nine patients (three pulmonary cases and six esophagus cases), who were treated with antibiotics. Rib fracture occurred in three patients (three pulmonary cases). Six patients experienced an anastomotic leak, and vocal cord palsy was found in ten patients in the esophageal group, who recovered after conservative treatment. There was no perioperative death. This is detailed in Table 2.

**Table 2:** Perioperative outcome.

	Pulmonary	Esophagus	Mediastinum
Docking time (min)	6.8 ± 4.9	7.7 ± 3.3	7.9 ± 4.3
Operation time (min)	Lobectomy 162 ± 59 Segmentectomy 194 ± 53 Wedge resection 69 ± 14 Sleeve lobectomy 212 ± 31	Esophageal cancer 391 ± 108 Robot console time 213 ± 98 Esophageal leiomyoma 142 ± 32	Anterior mediastinum 112 ± 78 Posterior mediastinum 82 ± 47
LN stations dissected	5.76 ± 2.23	12.2 ± 3.2	
Number of LNs			
Total LNs	15.5 ± 4.9	25.3 ± 6.5	
RRLN LNs	—	3.1 ± 1.9	
LRLN LNs	—	3.9 ± 2.3	
Thoracotomy conversions	0	2	0
Lung infection	3	6	
Vocal cord palsy	0	10	
Respiratory failure	0	3	0
Anastomotic fistula	/	6	—
Postoperative hospital stays (days)	6 ± 3	16 ± 9	5 ± 2
Tumor type	139 (78.1%)	3 (2.8%)	0
Adenocarcinoma	12 (6.7%)	103 (92.0%)	25 (59.5%)
Squamous cell carcinoma	27 (15.2%)	6 (5.2%)	
Other	Lung cancer (163)	Esophageal cancer (107)	17 (40.5%) /
Pathology T stage			
Tis	62 (38.0%)	3 (2.8%)	
T1	68 (41.7%)	36 (33.6%)	
T2	19 (11.7%)	43 (40.2%)	
T3	12 (7.4%)	22 (20.6%)	
T4	2 (1.2%)	3 (2.8%)	

LN, lymph nodes; LRLN, left recurrent laryngeal nerve; RRLN, right recurrent laryngeal nerve; Tis, tumor *in situ.*

## Discussion

Since the 1990s, thoracoscopic technology has been widely used and developed in thoracic surgery. Thoracoscopic surgery inevitably has its own limitations, such as limited visual information with two dimensions, restricted maneuverability of instruments, and an unsteady camera platform. The Da Vinci Surgical System (Intuitive Surgical, Sunnyvale, CA, USA) has revolutionized minimally invasive surgery by offering a more minimally invasive and precise approach to surgery (6). The Da Vinci Surgical System is composed of three parts: a surgeon control platform, a patient cart, and a three-dimensional view high-definition video cart. RATS approaches can be performed with a complete portal [described as robotic portal (RP) operation] or with the assistance of an access or utility incision [described as robotic-assisted (RA) operation] (7). There were different operative approaches between the RA and the RP operations. RA operations are usually a continuum from video-assisted thoracic surgery (VATS) to RATS for most surgeons. Additionally, either three or four robotic arms were used to perform RATS. Although a few surgeons used a completely port-based approach (RP operation: four robotic arms and no assistant port/incision) with the closed chest insufflated with CO_2_, RA operations with three robotic arms were more popularly used in RATS. In this article, all thoracic surgeries were performed through RA operations with three robotic arms, and a universal incision was also designed under this background, which may not be suitable for RP operations.

**Figure 3:**
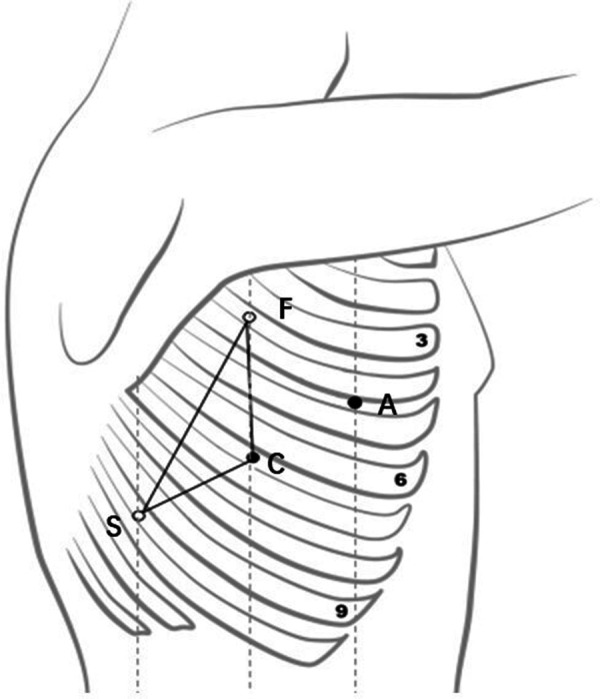
Robotic arm placement (an isosceles triangle). C, camera port; A, assistant port; F, first robotic arm; S, second robotic arm).

The selection of robot-assisted surgical incision should follow certain principles, which could ensure that the instruments are flexible in the thoracic cavity during the surgery and do not interfere with one another. The general principle of surgical incision selection is that the distance between the camera port and the incisions for the first robotic arm and the second robotic arm should be more than 8 cm. The triangle target principle for the placement of trocars during VATS was first named by Sasaki et al. (7), and these principles should be followed during RATS. According to our experience, the incision for the camera port, which serves as the vertex of the isosceles triangle, and its connection with the incisions for the first and second robotic arms form an isosceles triangle. The selection for the assistant port should not be placed in the isosceles triangle to the greatest extent, as shown in Figure 3. There are many types of thoracic surgery, including pulmonary, esophageal, and mediastinum tumor surgery. While the thoracic cavity is large and requires extensive coverage, different operations have different emphases and different exposures of the surgical area. For example, esophageal surgery is mainly located in the posterior mediastinum, and pulmonary surgery mainly requires wide exposure from the lung hilum to the tracheal carina and superior mediastinum, while mediastinal tumor surgery requires different exposure parts according to different lesion locations. Therefore, while selecting the robot-assisted surgical incision, different surgeons usually have different choices (6, 8–22), as given in Table 3. Even for pulmonary surgery, at present, there are still a variety of robot-assisted surgical incision selections (6, 9, 14–17, 19, 20, 22). Oh et al. (23) summarized robotic port placement, which was used by high-volume thoracic surgeons in the United States who performed robot-assisted lobectomy, and they found that the precise locations of the robotic ports were heterogeneous for each lobectomy. The most common locations for camera and instrument trocars were the seventh and eighth interspaces for all types of lobectomies. The placement of trocars for robot-assisted lobectomy was flexible and based on the clinician's experience or the unique anatomic issues of a specific patient. These incisions are suitable only for pulmonary or esophageal surgery and mediastinal tumor surgery, and they do not constitute a universal incision for RATS.

**Table 3:** A brief summary of the incisions for robot-assisted thoracic surgery.

	Camera port	The first robotic arm	The second robotic arm	The assistant port	The third robotic arm
Pulmonary					
Veronesi et al. (6)	7th ICS, MAL	4th ICS, AAL	8th ICS, PAL	—	7th ICS, ISL
Pardolesi etal. (16)	7th/8th ICS, MAL	8th ICS, PAL	Posteriorly in the AT	4th/5th ICS, AAL	
Zhao et al. (22)	8th ICS, MAL	7th ICS, PAL	5th ICS, AAL	9th/10th ICS, PAL	—
Li et al. (14)	8th ICS, PAL	7th ICS, MAL	9th ICS, ISL	4th ICS, AAL	
Esophageal surgery					
Kim et al. (13)	8th ICS, ISL	10th ICS, ISL	6th ICS,PAL	7th ICS, MAL	
Kingma et al. (10)	6th ICS between PAL and SL	10th ICS	8th ICS between PAL and ISL	5th ICS, PAL	4th ICS between PAL and ISL
Anterior mediastinum					
Surgery					
Augustin et al. (8)	5th ICS, AAL	3rd ICS, AAL	5th ICS, MCL	5th ICS, MAL	
Kamel et al. (12)	6th ICS, PAL	3rd ICS, AAL	5th ICS, AAL		

ICS, intercostal space; AAL, anterior axillary line; MAL, midaxillary line; PAL, posterior axillary line; MCL, midclavicular line; ISL, infrascapular line; AT, auscultatory triangle.

As there are many types of thoracic surgery, the variety of incision selection presents some difficulties to the chief surgeon, especially for a beginner in carrying out RATS. Robot-assisted surgeons are skilled in thoracoscopic surgery, and the learning curve of robot-assisted surgery is much shorter than that of thoracoscopic surgery (24–26). Based on the practice, exploration, and summary of more than 300 cases of robot-assisted surgery, the concept of universal thoracic incision in robot-assisted surgery was proposed. The incision for the camera port was placed at the midaxillary line in the sixth ICS. The incisions for the first robotic arm were placed at the midaxillary line in the third ICS, the incision for the second robotic arm was placed at the subscapular line in the ninth ICS, and the assistant port was placed at the anterior axillary line in the fourth ICS. This incision is applicable to all lung, esophageal, and posterior mediastinal tumor surgeries. For anterosuperior mediastinal tumors, the incision for the second robotic arm was adjusted at the anterior axillary line in the sixth ICS. If necessary, the assistant port could be adjusted at the posterior axillary line in the eighth ICS. The distance between the incisions for the first robotic arm and the second robotic arm from the camera port should be kept a palm wide (approximately 8 cm). The incision for the camera port should be made first in practice, and the remaining incisions are placed under direct visualization to guarantee the incision within the thoracic cavity. Blind operations are strictly forbidden to avoid injury to the diaphragm or entry into the abdominal cavity.

Among the 342 cases of clinical surgery, there were 107 cases of esophageal cancer surgery (McKeown approach), 5 cases of esophageal leiomyoma, 178 cases of pulmonary surgery, and 52 cases of mediastinal tumor surgery. Two cases of early surgery were transferred to VATS with a small incision for serious chest adhesion, and the remaining cases were not transferred to VATS or thoracotomy. All the surgeries were successfully completed, with no deaths during the perioperative period or one month after surgery. In our previous study (27, 28), the safety and feasibility of robot-assisted minimally invasive esophagectomy (RAMIE) compared with video-assisted minimally invasive esophagectomy (VAMIE) and RATS lobectomy compared uniportal VATS lobectomy were evaluated individually. There was no significant difference in the rate of overall complications between RATS and VATS. Compared with VATS, a greater number of lymph nodes harvested were found in RAMIE and RATS lobectomy. There have been several reports on the advantages of robots in lymph node dissection (14, 18). RAMIE could retrieve more thoracic lymph nodes along the recurrent laryngeal nerve areas. Park et al. (18) reported a mean total of 43.5 ± 1.4 retrieved lymph nodes. Although the number of lymph nodes harvested in the present study was smaller, there was also statistical significance between the RAMIE and the VAMIE groups in our previous study (28).

The initial design of this robot-assisted thoracic incision gave priority consideration to esophageal surgery, and nearly all 40 robot-assisted surgery cases during the early period were patients with esophageal tumor. Based on robot-assisted surgery experience, it was found in subsequent lung surgical explorations that the universal incision for pulmonary surgery also had very good exposure and operation effects. Thus, lobectomy and segmentectomy were carried out afterward. The assistant port was placed at the anterior axillary line in the fourth ICS, which fits the operation habits of VATS, especially with regard to the exposure in uniportal VATS and the placement of a linear cut stapler. The assistant with uniportal VATS experience can conveniently operate on the table and shorten the operating time, thus ensuring skilled coordination between the assistant and the chief surgeon. The location of the assistant port in the anterior chest wall is also conducive to rapid thoracotomy in cases of emergency massive bleeding during surgery (although we have not encountered such situations). Two cases of esophageal cancer complicated with nodules in the upper lobe of the right lung successfully underwent RATS through this surgical incision. After the separation of the esophagus and lymph node dissection, resection of the right upper lobe was completed, which further reflected the superiority of the universal surgical incision. The EndoWrist® in the da Vinci system is superior to the human wrist, as it is flexible in all directions. There are a few reports about the cases of RATS sleeve or double-sleeve lobectomy for central-type lung cancer (29–32). Due to the small number of surgical cases, only three cases of bronchial sleeve resection of the pulmonary lobe (one case for the right upper pulmonary lobe and two cases for the left upper pulmonary lobe) were completed. The 3-0 prolene sutures (ETHICON 24 mm 1/2c, USA) in a continuous way were used to perform the bronchial sleeve resection. It was found to be more successful for intraoperative sutures than for thoracoscopic sutures, which showed a great advantage over the former.

For the anterior mediastinal tumor, the lesion is located in the substernal part with a narrow space. When the lesion is too large, its exposure under the thoracoscope is poorer. The advantages of robot-assisted surgery are obvious for fine operations within such a narrow space. For the anterior mediastinal tumor, the incision for the second robotic arm is moved to the anterior chest wall, and the anterior superior mediastinal tumors with lesions below 3 cm can be completed independently without an assistant port, while solid tumors with lesions above 3 cm often require an additional assistant port to enhance the exposure of the operative field. The assistant port can be placed at the posterior axillary line in the eighth ICS. The largest anterior superior mediastinal tumor (solid thymoma) was completely excised through this incision, which was nearly 8 cm in diameter, avoiding thoracotomy or sternum splitting and minimizing trauma to the patient.

Good robotic surgical incision design is the premise of a successful operation and can display robot platform advantages. A relatively simple and fixed general surgical incision, good intraoperative exposure, and quick and skilled cooperation of the assistant can reduce the difficulty in RATS for the surgeon and shorten the learning curve of robot-assisted surgery. Our preliminary experience suggests that universal robot incisions are feasible for esophageal, lung, and most mediastinal tumors. The proposal of a universal robot incision provides a simple and easy incision design for an increasing number of thoracic surgeons to ensure the smooth and successful development of RATS.

This study also has some limitations. Due to the small number of surgical cases, there is no relevant experience in the Ivor Lewis approach for esophageal cancer and pulmonary artery plasty, and as a result, only three cases of bronchial sleeve resection have been completed. In addition, this paper included data from only two surgical centers. More surgical centers need to try and verify the safety and convenience of this universal incision. However, this universal incision for RATS has great value as it may guide standardized port placement, which would be important for the learner and the instructor.

## Data Availability

The raw data supporting the conclusions of this article will be made available by the authors without undue reservation.
